# Low HDL-C/ApoA-I index is associated with cardiometabolic risk factors and coronary artery calcium: a sub-analysis of the genetics of atherosclerotic disease (GEA) study

**DOI:** 10.1186/s12902-024-01642-0

**Published:** 2024-07-11

**Authors:** Guillermo Celestino Cardoso-Saldaña, Neftali Eduardo Antonio-Villa, María del Rocío Martínez-Alvarado, María del Carmen González-Salazar, Rosalinda Posadas-Sánchez

**Affiliations:** Department of Endocrinology, National Institute of Cardiology Ignacio Chávez, Juan Badiano No. 1, Col Sección XVI, CP 14080 Tlalpan, México City, Mexico

**Keywords:** HDL-C/Apo-AI index, Coronary artery calcium score, Heart disease biomarker

## Abstract

**Background:**

The high-density lipoprotein cholesterol to apolipoprotein A-I index (HDL-C/ApoA-I) may be practical and useful in clinical practice as a marker of atherosclerosis. This study aimed to investigate the association between the HDL-C/ApoA-I index with cardiometabolic risk factors and subclinical atherosclerosis.

**Methods:**

In this cross-sectional sub-analysis of the GEA study, 1,363 individuals, women (51.3%) and men (48.7%) between 20 and 75 years old, without coronary heart disease or diabetes mellitus were included. We defined an adverse cardiometabolic profile as excess adipose tissue metrics, non-alcoholic liver fat measured by non-contrasted tomography, metabolic syndrome, dyslipidemias, and insulin resistance. The population was stratified by quartiles of the HDL-C/Apo-AI index, and its dose-relationship associations were analysed using Tobit regression, binomial, and multinomial logistic regression analysis.

**Results:**

Body mass index, visceral and pericardial fat, metabolic syndrome, fatty liver, high blood pressure, and CAC were inversely associated with the HDL-C/ApoA-I index. The CAC > 0 prevalence was higher in quartile 1 (29.2%) than in the last quartile (22%) of HDL-C/ApoA-I index (*p* = 0.035). The probability of having CAC > 0 was higher when the HDL-C/ApoA-I index was less than 0.28 (*p* < 0.001). This association was independent of classical coronary risk factors, visceral and pericardial fat measurements.

**Conclusion:**

The HDL-C/ApoA-I index is inversely associated with an adverse cardiometabolic profile and CAC score, making it a potentially useful and practical biomarker of coronary atherosclerosis. Overall, these findings suggest that the HDL-C/ApoA-I index could be useful for evaluating the probability of having higher cardiometabolic risk factors and subclinical atherosclerosis in adults without CAD.

**Supplementary Information:**

The online version contains supplementary material available at 10.1186/s12902-024-01642-0.

## Background

Epidemiological and clinical studies have shown that low concentrations of high-density lipoprotein cholesterol (HDL-C) are associated with an increased risk of coronary artery disease (CAD) [1–4]. However, in other studies, normal and even high concentrations of HDL-C are associated with CAD [5, 6]. Moreover, several clinical [7–9] and genetic studies [4] have not been able to demonstrate the cardioprotective role of high HDL-C levels. Therefore, the role of high-density lipoprotein in the pathogenesis and progression of CAD is controversial. This may be explained by the size and particle number that could play a role in the development of atherosclerotic plaque [10–12]. The HDLs can be classified mainly into two classes: HDL_2_ and HDL_3,_ and into several sub-groups [13, 14]. The methods to determine the size and composition of HDL sub-classes require specialized techniques and equipment like ultracentrifugation, nuclear magnetic resonance, or X-ray crystallography. Although these methods are accurate and precise, they require expensive equipment, specialized technical training, larger sample volume, and longer time to obtain the result, so their application in clinical practice is still far from being used in clinical laboratories and medical practice. [15]. The use of practical alternatives to determine the HDL size and composition, although clinically useful, has been a challenge and area of opportunity. A previous study demonstrated that high HDL-C/ApoA-I index is linked with functional cholesterol-rich particles and has a clinical association with a reduction in atherosclerotic progression. Therefore, the HDL-C/ApoA-I index could be a marker of HDL composition and function in cardiometabolic risk factors. [16] Furthermore, diverse studies show that the HDL-C/ApoA-I index correlates with the diameter of HDL measured by nuclear magnetic resonance in patients with different genetic and metabolic profiles [17]. In clinical practice, the risk of coronary artery disease is classified according to the number and severity of the patient’s risk factors; however, in the general population, the risk of atherosclerosis is frequently classified as intermediate or low. In these cases, the HDL-C/Apo-AI index, and beyond the HDL cholesterol content, can be used as a surrogate of HDL size and functionality to optimize the risk assessment and justify an appropriate treatment. However, it is unknown whether the HDL-C/ApoA-I index could be associated with subclinical atherosclerosis evaluated by coronary artery calcium score (CAC) in adults with asymptomatic CAD. Therefore, in this sub-analysis of the GEA study, we aimed to investigate the association between the HDL-C/ApoA-I index with cardiometabolic risk factors and subclinical atherosclerosis, as measured by CAC score using computed tomography (CT).

## Materials and methods

### Study design and subjects

This study represents a sub-analysis of 1,363 from 1,600 subjects without CAD enrolled in the Genetics of Atherosclerotic Disease (GEA) study. Complete methods and preliminary reports have been described elsewhere [18, 19]. Briefly, the GEA study was designed to investigate the genetic basis of CAD and the relationship between traditional and emerging risk factors for atherosclerosis in the Mexican population. The volunteers recruited were women and men, selected from the donors who attended the National Institute of Cardiology Ignacio Chavez or recruited by invitation among the population attending several health community clinics in the metropolitan area of Mexico City. For this analysis, we selected individuals between 20 and 75 years old, without a personal or family history of premature CAD or diabetes, no clinical evidence of kidney disease (serum creatinine < 0.132 mmol/L), or liver disease (viral or drug-induced hepatitis). The study protocol (No. 09-646) was approved by National Institute of Cardiology Ethics Committee and the study protocol conforms to the ethical guidelines of the 1975 Declaration of Helsinki. All participants signed an informed consent letter.

### Anthropometrical, clinical, and biochemical measurements

Validated questionnaires were applied to obtain sociodemographic, personal history of cardiovascular risk factors, physical activity, alcohol consumption, and use of pharmaceutical drugs. Weight in kilograms (kg) and height in centimeters (cm) were obtained using a calibrated scale and a wall stadiometer. Body mass index (BMI) was calculated using the formula: weight (kg) / height (m^2^). Waist circumference was measured with a fiberglass metric tape at the midpoint of the distance between the bottom of the last rib and the iliac crest to the nearest 0.5 cm. Systolic blood pressure (SBP) and diastolic blood pressure (DBP) were measured three times in a sitting position. The average of the last two measurements was used for the analysis. Physical activity was quantified using the Baecke´s questionnaire [20]. The total activity was obtained by adding the activity indices at work, during exercise, and during leisure time.

### Biochemical determinations

Venous blood samples were collected after 10 h of fasting and 20 min in a sitting position. Plasma glucose, total cholesterol (TC), triglycerides (TG), and high-density lipoprotein cholesterol (HDL-C), were assayed using enzymatic colorimetric methods, and ApoA-I and Apo-B100 plasma levels were determined by immunoturbidimetry, (Roche/Hitachi, Germany) on a Hitachi analyser 902 (Hitachi LTD, Tokyo, Japan). Low-density lipoprotein cholesterol (LDL-C) was calculated using the Friedewald´s formula [21]. The reproducibility and precision of lipid and lipoprotein determinations were certified by the Center for Disease Control and Prevention through the Lipid Standardization Program (LSP-CDC, Atlanta, GA, USA). The intra- and inter-assay coefficients of variation were less than 3%. Serum insulin concentration was determined by radioimmunoassay (Human Insulin RIA Kit; Millipore, Cat. HI-14k, St Charles, Missouri USA). Insulin resistance was estimated with the homeostatic model of insulin resistance (HOMA-IR: Insulin IU/mL X Glucose mmol/22.5) [22]. Intra- and inter-assay coefficients of variation were 2.1 and 6.8%, respectively.

### Computed tomography assessment

Non-contrasted computed tomography (CT) is a validated method to quantify abdominal, visceral, subcutaneous, and pericardial fat depots and the coronary artery calcium [23–25]. In the present study, abdominal CT was performed through a 64-channel detector helical tomography system (Somaton Sensation, Siemens, Malvern, PA USA). To quantify the total abdominal fat (TAF), subcutaneous abdominal fat (SAF), and visceral abdominal fat (VAF), the scan were obtained without contrast as described by Kvist H. et al. [25], the pericardial fat volume (PCF) as described by Dey et al. [26] and liver to spleen attenuation ratio (L/SAR) as described by Longo R. et al. [27]. We estimated the CAC score using the Agatston method [23]. The intra-observed variability for CAC was analysed in 20 random cases, the correlation coefficient was 0.99 (*p* < 0.001).

### Cardiometabolic risk factors and subclinical atherosclerosis definition

Our study was centered on two main outcomes: the association of HDL-C/ApoA I ratio with cardiometabolic risk factors and subclinical atherosclerosis.

I- Cardiometabolic risk factors: Cut-off points to define insulin resistance and excess adipose tissue were obtained on 316 individuals (131 men and 185 women) with the following characteristics: BMI < 30 kg/m2, without diabetes mellitus, dyslipidaemia, or arterial hypertension.

- Insulin resistance: Defined if the HOMA-IR index was greater than 3.44 in men and 3.45 in women.
- Arterial hypertension: Defined when systolic blood pressure was ≥ 140 mmHg or diastolic blood pressure was ≥ 90 mmHg, and/or use of antihypertensive drugs.
- Adipose tissue thresholds: The 75th percentile was used to define high values for VAF 152.5 cm^2^ in men and 121 cm^2^ in women, for SAF 221 cm^2^ in men and 320.5 cm^2^ in women and 58.6 cm^2^ for PCF.
- Overweight and obesity: Defined as a BMI between 25 and 29.9 or greater than 30 kg/m^2^, respectively [28].
- Dyslipidaemia profile: The presence of dyslipidaemia was defined according to the criteria of the National Cholesterol Education Program Adult Treatment Panel III (NCEP-ATP III) [29] as: hypercholesterolemia (TC ≥ 5.2 mmol/L or LDL-C ≥ 4.2 mmol/L), hypertriglyceridemia (TG ≥ 1.69 mmol/L), hypoalphalipoproteinemia (HDL-C < 1.104 mmol/L in men or < 1.3 mmol/L in women).
- Non-alcoholic fatty liver disease (NAFLD): Defined as liver to spleen attenuation ratio (L/SAR) < 1.0 [27].
- Metabolic syndrome: Defined according to the NCEP-ATP III criteria as having central obesity (waist circumference ≥ 90 cm in males and ≥ 80 cm in females), elevated triglycerides (> 150 mg/dL) or treatment for elevated TG, reduced HDL-cholesterol < 1.104 mmol/L in men or < 1.3 mmol/L in women), elevated blood pressure (> 130 mmHg systolic blood pressure or > 85 mmHg diastolic blood pressure) or medical treatment for hypertension, and elevated fasting glucose (≥ 5.55 mmol/L).

II- Subclinical atherosclerosis: This outcome was defined as the presence of a CAC score > 0 HU. To further evaluate the impact of HDL-C/ApoA-I index and subclinical atherosclerosis, we categorized identified subjects with CAC ≥ 10 HU and CAC ≥ 100 HU. [30]

### Statistical analysis

Continuous variables with normal distribution were expressed as mean ± SD, and categorical variables as percentages. The variables with asymmetric distribution were analysed using parametric statistics. The CAC score was modified as CAC + 1 and then logarithmically transformed to have a better symmetric distribution. The population was then stratified by HDL-C/Apo-AI index quartiles (Q) (Q1: 0.10 to 0.28, Q2: 0.29 to 0.33, Q3: 0.34 to 0.38, Q4: 0.39 to 0.69). A chi-square test was applied to compare the prevalence of CAD risk factors between quartiles. Variables with normal distribution were compared with ANOVA and interquartile differences were determined using the Tuckey’s test. A *p* < 0.05 value was considered statistically significant. The analyses were performed with the statistical package SPSS v15.0 (SPSS Chicago, II), and figures were obtained with R (Version 4.2.1).

#### Evaluation of HDL-C/Apo-AI index and cardiometabolic risk factors

First, we evaluate the dose-relationship correlation between HDL-C/Apo-AI index and continuous markers of cardiometabolic risk factors using the Spearman correlation coefficient at a 95% confidence interval. We further fitted linear regression models adjusted for age, sex, BMI, TG, LDL-C, HOMA-IR, L/SAR, physical activity, and smoking, except for the models in which these variables are included as outcomes. Then, we estimate the prevalence of cardiometabolic risk factors and subclinical atherosclerosis with a 95% confidence interval using the Clopper-Pearson approach stratified by HDL-C/Apo-AI index quartiles. To evaluate a trend association with HDL-C/Apo-AI index quartiles and coronary risk factors, we fitted binomial logistic regression models with cardiometabolic risk factors as the dependent variable and HDL-C/Apo-AI index quartiles as the independent variable adjusted for the same covariates used in linear regression models.

#### Association of HDL-C/Apo-AI index with subclinical atherosclerosis

In asymptomatic and healthy subjects, the atherosclerotic burden is minimal. Therefore, the CAC score tends to be zero, leading to a left-censoring bias. Additionally, values equal to zero lead to a wide dispersion variance in most generalized linear models. Therefore, to analyse the linear relationship between the HDL-C/Apo-AI index and CAC score, we fitted Tobit regression models. Then, as a sensitivity analysis, we evaluate the association of the HDL-C/Apo-AI index and CAC categories (CAC > 10 HU and CAC ≥ 100 HU) using multinomial logistic regression models. Both Tobit and multinomial regression models were adjusted as follows: Model 1 was evaluated as an univariable adjustment. Model 2 was adjusted for BMI, TC, TG, LDL-C, HOMA-IR, and NAFLD. Finally, Model 3 included the variables in Model 2 plus GAV, sedentary lifestyle, and smoking habit. The goodness of fit was evaluated using the Bayesian Information Criteria (BIC).

## Results

### Characterization amongst HDL-C/ApoA-I index quartiles

Our study included a sample of 1,363 subjects (85.2% from baseline), 51.3% women and 48.7% men, with a mean age of 52.6 ± 9.3 years, BMI of 29.3 ± 4.3, VAF of 153.3 ± 63.1 cm2, SAF of 294.4 ± 112.2 cm2, PCF of 46.3 ± 24.3 cm3, and CAC of 22.4 ± 105.6 HU. The anthropometric, clinical, and biochemical characteristics stratified by quartiles of HDL-C/ApoA-I index are shown in Table 1. Briefly, participants in the first quartile of HDL-C/ApoA-I index had higher weight, BMI, systolic and diastolic blood pressure, VAF, and PCF, with a progressively decreasing trend observed across the quartiles. The mean values of SAF and Ln-CAC + 1 were also lower in the first than in the last quartile. The HDL-C levels were lower, while concentrations of LDL-C, TG, Apo-AI, glucose, insulin, and HOMA-IR were higher among subjects in the first quartile compared with the fourth.

**Table 1:** Anthropometric, physiological and biochemical characteristics by quartiles of HDL-C/ApoA-I index

	Quartiles of HDL-C/Apo-AI index				
	Q1 (0.10-0.288)	Q2 (0.29–0.337)	Q3 (0.34–0.389)	Q4 (0.39–0.69)	*p* value
Anthropometry					
N (% Women)	320 (42.4)	346 (47.4)	345 (53.3)	352 (62.4)	< 0.001
Age (years)	52.05 ± 9.31	52.25 ± 9.01	52.42 ± 9.70	53.67 ± 9.23	0.098
Weight (kg)	77.83 ± 13.94	74.97 ± 13.62^a*^	73.92 ± 13.75 ^b*^	70.06 ± 13.41^(c, d, e)†^	< 0.001
BMI (kg/m^2^)	29.44 ± 4.00	28.51 ± 4.03^a*^	28.38 ± 4.52 ^b*^	27.06 ± 4.27 ^(c, d, e)†^	< 0.001
WC, (cm)	97.3 ± 10.6	95.6 ± 10.9	93.9 ± 11.3	90.8 ± 12.1 ^(b, e)†^	< 0.001
SBP (mmHg)	118.93 ± 17.05	114.71 ± 15.52^a*^	116.97 ± 18.36	114.10 ± 16.83^c†^	0.001
DBP (mmHg)	73.77 ± 9.54	71.17 ± 8.54^a*^	72.26 ± 10.53	70.86 ± 9.06^c*^	<0.001
Physical activity score	7.85 ± 1.3	7.87 ± 1.20	7.78 ± 1.20	7.94 ± 1.23	0.434
Fat and CAC depots					
VAF (cm^2^)	169.96 ± 62.49	158.10 ± 57.34^a*^	149.01 ± 65.04 ^b†^	137.51 ± 63.38 ^(c, d, e) †^	< 0.001
PCF (cm^2^)	55.26 ± 28.8	49.18 ± 23.5^a*^	45.76 ± 21.9 ^b*†^	36.34 ± 18.5 ^(c, d, e)†^	< 0.001
SAF (cm^2^)	297.26 ± 103.63	294.54 ± 112.17	300.50 ± 120.83	285.14 ± 111.39	0.262
L/SAR	0.981 ± 0.25	1.034 ± 0.25	1.073 ± 0.220	1.115 ± 0.247	< 0.001
CAC, (Ln UH)	0.814 ± 1.59	0.770 ± 1.62	0.753 ± 1.63	0.664 ± 1.50	0.442
Blood Chemistry					
TC, (mmol/L)	4.96 ± 1.03	4.98 ± 0.98	5.09 ± 0.87	4.99 ± 0.90	0.269
LDL-C (mmol/L)	3.04 ± 0.88	3.12 ± 0.83	3.20 ± 0.77	3.01 ± 0.80 ^f*^	0.014
HDL-C (mmol/L)	0.93 ± 0.24	1.02± 0.26^a†^	1.26 ± 0.29 ^(b, d)†^	1.47 ± 0.37 ^(c, e, f)†^	< 0.001
TG (mmol/L)	2.63 ± 1.98	2.00 ± 0.88^a†^	1.70 ± 0.70 ^b†, d*^	1.40 ± 0.60 ^c†, e, f*^	< 0.001
Apo-AI (g/L)	1.51 ± 0.46	1.37 ± 0.32a†	1.34 ± 0.31b ^†^	1.29 ± 0.29 ^c†, e*^	< 0.001
Glucose (mmol/L)	5.1 ± 0.54	4.98 ± 0.5^a+^	4.9 ± 0.53 ^b*^	4.9 ± 0.47^c†^	< 0.001
Insulin (IU/mL)	22.1 ± 11.0	19.5 ± 10.3^a+^	18.4 ± 9.5 ^b†^	16.2 ± 8.0 ^(c,e)†, f*^	< 0.001
HOMA-IR	5.0 ± 2.7	4.3 ± 2.5^a+^	4.1 ± 2.5 ^b+^	3.5 ± 1.9 ^(c, e)+, f*^	< 0.001

The values express the mean ± SD. BMI: Body Mass Index, WC: Waist Circumference, SBP: Systolic Blood Pressure, DBP: Diastolic Blood Pressure, VAF: Visceral Abdominal Fat, SAF: Subcutaneous Abdominal Fat, PCF: Pericardial Fat Volume. L/SAR: Liver to spleen attenuation ratio, CT: Total cholesterol, LDL-C: low-density lipoprotein cholesterol, HDL-C: High density lipoprotein cholesterol, TG: Triglycerides and HOMA-IR: Homeostatic model of insulin resistance. Differences between quartiles (Q) were obtained using the Tuckey test. *p < 0.05; †*p* ≤ 0.001: a: QC1vs.Q2; b: Q1vs.Q3; c: Q4; d: Q2vs.Q3; e: Q2 vs. Q4, f: Q3vs.C4

### Association HDL-C/ApoA-I index with cardiometabolic risk factors

**Fig. 1:**
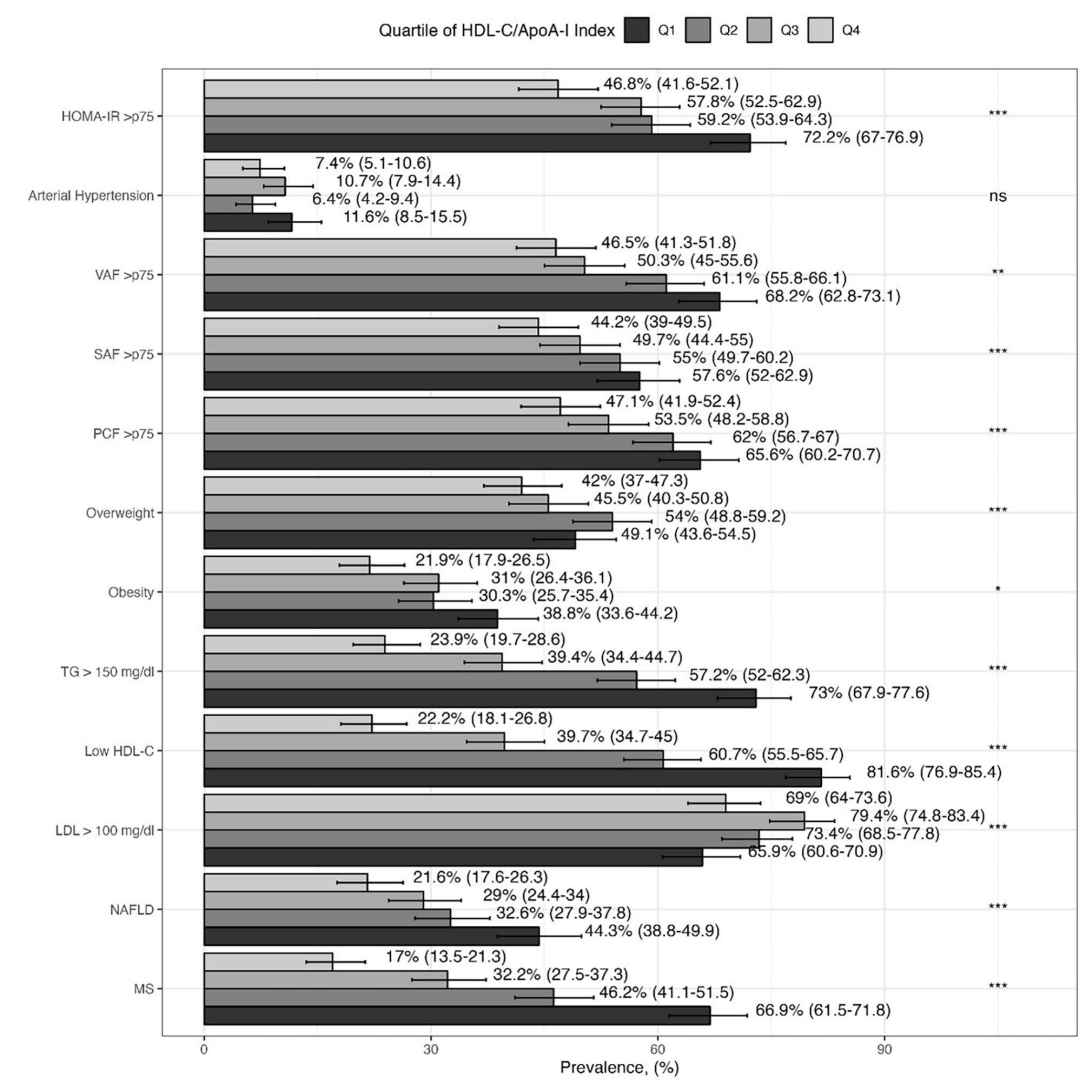
Cardiometabolic risk factors prevalence by quartile of the HDL-C/ApoA-I index in subjects without CAD. Data represent the percentage and (95% CI). *HDL-C < 50 in women and < 40 in men, TG: triglycerides, MS: Metabolic Syndrome, PCF: Pericardial Fat Volume, VAF: Visceral Abdominal Fat and SAF: Subcutaneous Abdominal Fat. Notations: *** =*p* < 0.001; ** =*p* < 0.01; * =*p* < 0.05; ns = non-significant

We observed that HDL-C/ApoA-I index was inversely correlated with TG (*r* = -0.437, 95% CI: -0.478 to -0.392, *p* < 0.001), HOMA-IR (*r* = -0.225, 95% CI: -0.275 to -0.173, *p* < 0.001), PCF (*r*= -0.288, 95% CI: -0.344 to -0.228, *p* < 0.001), the VAF (*r* = -0.220, 95% CI: -0.271 to -0.168, *p* < 0.0001) and positively correlated with the L/SAR (*r* = 0.212, 95% CI: 0.159 to 0.262, *p* < 0.0001). Complete correlation coefficients are presented in Supplementary Table 1. To confirm these correlations, linear regressions were fitted and showed that the HDL-C/ApoA-I index was positively associated with age and HDL-C and negatively associated with waist circumference, BMI, SBP, DBP, TG, Non-HDL-C, glucose, insulin, HOMA-IR, VAF, PCF, and L/SAR (Supplementary Table 2). Furthermore, participants in Q1 of the HDL-C/ApoA-I index had a higher prevalence of excess adipose tissue, NAFLD, dyslipidemia, MS, and HOMA-IR compared with subjects with upper quartiles (Fig. 1). These trends were confirmed using logistic regression models. After considering the adjustment for confounders, participants in Q1 of HDL-C/ApoA-I index had a higher probability of having hypertriglyceridemia (OR: 7.31, 95% CI: 5.03–10.7, *p* < 0.001), hypoalphalipoproteinemia (OR: 8.40, 95% CI: 5.60–12.7, *p* < 0.001), NAFLD (OR: 1.72, 95% CI: 1.15–2.58, *p* = 0.008) and MS (OR: 3.10, 95% CI: 1.97–4.94, *p* < 0.001) (Supplementary Table 3).

### Association HDL-C/ApoA-I index with subclinical atherosclerosis

**Fig. 2:**
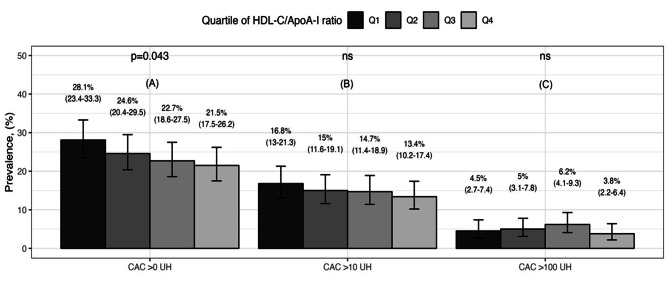
Prevalence of CAC score by quartiles of HDL-C/Apo-AI index. Data represent the percentage and (95% CI). **(A)** Prevalence of CAC > 0, **(B)** CAC > 10 and **(C)** CAC > 100 HU

In the overall sample, the prevalence of CAC > 0 HU was 24% (95% CI: 21.9–26.5%). We observe that the HDL-C/ApoA-I index negatively correlated with ln CAC + 1 (*r*= -0.058, 95% CI: -0.111 to -0.0043, *p* = 0.034). Furthermore, there was an overall difference of CAC > 0 HU among subjects with Quartile 1 (28.1%, 95% CI: 23.4–33.3%) compared with subjects in Quartile 4 (21.5%, 95%: 17.5–26.2%). We observed a similar trend when the sample was stratified by CAC > 10 HU and CAC > 100 HU, although the latter has non statistical significance (Fig. 2).

Then, we tested the independent associations of CAC score and HDL/Apo-A1 index using Tobit regression. This analysis shows that for each unit that the HDL-C/Apo-AI index increases, the CAC score decreases by -2.71 HU (95% CI: -5.168 to -0.261, *p* < 0.03). However, after adjusting for confounding variables, models 2 and 3, the CAC score decreases by -2.429 HU (95% CI: -5.271 to 0.412, *p* = 0.093) and − 2.464 HU (95% CI: -5.262 to 0.377, *p* = 0.084) (Table 2**)**.

**Table 2:** Tobit regression models to predict tomographic coronary calcium score using HDL-C/ApoA1 index

Parameter	Model	B Coefficient	95% CI	Z-Value	*p*
	1 BIC: 5290.764	-2.714	-5.168, -0.261	-2.169	0.030
HDL-C/ApoA-I index	2 BIC: 5146.752	-2.429	-5.271, 0.412	-1.676	0.093
	3 BIC: 5089.082	-2.464	-5.262, 0.377	-1.726	0.084

Model 1: Unadjusted; Model 2: Adjusted for BMI, TG, LDL-C, HOMA-IR, L/SAR;

Model 3: Model 2 + GAV, physical activity, and smoking

BIC = Bayesian information criteria

**Fig. 3:**
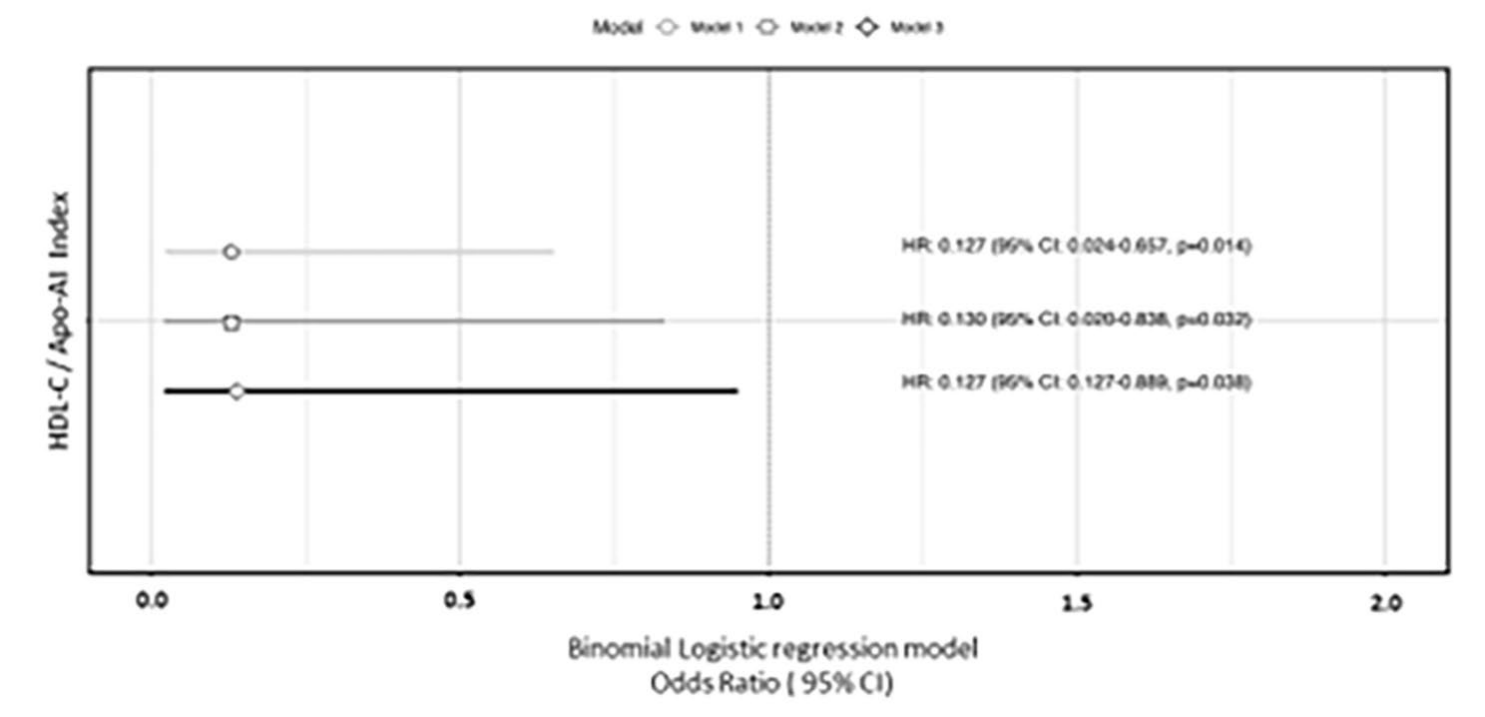
Multivariate regression analysis between the HDL-C/ Apo-AI index and CAC > 0 HU. Model 1: Not adjusted, Model 2: Adjusted for BMI, TG, LDL-C, HOMA-IR, non-alcoholic fatty liver. Model 3: Model 2 plus GAV, physical activity and smoking

We then fitted binomial logistic regression analysis between CAC > 0 HU with HDL-C/Apo-AI index. This analysis showed that higher values of HDL-C/ApoA-I index were associated with a lower probability of having a CAC score > 0 HU (OR: 0.127, IC 95%: 0.018–0.889, *p* = 0.038; Fig. 3).

Finally, we fitted multinomial logistic regression analyses between HDL-C/ApoA-I index with CAC > 10 HU and CAC > 100 HU. We observed that after adjustment, higher HDL/Apo-A1 ratio values were negatively associated only with CAC score > 10 (Table 3).

**Table 3:** Multinomial logistic regression models to predict tomographic coronary calcium score using HDL-C/ApoA-I index

Predictor	Model	Outcome	β Coefficient	Odds ratio	95% CI	*p*
	1	CAC > 10	-2.375	0.092	0.015–0.564	0.010
		CAC ≥ 100	-0.874	0.417	0.016–10.80	0.598
HDL-C/ApoA-I ratio	2	CAC > 10	-2.593	0.074	0.010–0.593	0.014
		CAC ≥ 100	0.853	2.347	0.055–99.87	0.655
	3	CAC > 10	-2.675	0.068	0.009–0.548	0.012
		CAC ≥ 100	0.780	2.182	0.551–8.633	0.266

CAC: Coronary artery calcium. Model 1: Unadjusted; Model 2: Adjusted for BMI, TC, TG, LDL-C, HOMA-IR, L/SAR; Model 3: Model 2 + GAV, physical activity and smoking

## Discussion

In this sub-analysis of the GEA study, we showed that the HDL-C/ApoA-I index was inversely associated with adverse cardiometabolic profile and subclinical atherosclerosis in subjects without a personal or familial CAD history. Stratifying by HDL-C/ApoA-I quartiles, we demonstrate that subjects in the lower quartile of the HDL-C/Apo-AI index had a higher probability of subclinical atherosclerosis. Moreover, the association with the HDL-C/Apo-AI index was sustained only for subjects with CAC > 10 HU, which suggests that this index could help identify subjects with low atherosclerosis.

Our findings agree with diverse results previously reported in patients with ischemic heart disease [16] and with dyslipidaemias [31, 32]. There is consistent evidence that the HDL-C/ApoA-I index is an optimal surrogate of HDL size. The results of this study support the hypothesis that the size of HDL obtained with the HDL-C/ApoA-I index can be considered integrative measures of the heterogeneity of these lipoproteins [33] that also allow a practical evaluation of subclinical atherosclerosis risk. The inverse relationship between the HDL-C/Apo A-I ratio and the CAC score could be due to the effect of small HDL (HDL3), which stimulates the transcription of genes for osteopontin and bone morphogenic protein-2 related to coronary calcification [34]. Furthermore, HDLs induce the interaction between osteoprotegerin and the receptor activator of nuclear factor NFκB during the process of aortic valve calcification [35]. It could be possible that by a similar process, there may be smaller HDLs participate in the calcification of the coronary arteries [36]. On the other hand, the greater volume of pericardial fat in subjects with an HDL-C/ApoA-I index less than 0.28 suggests that the inflammatory molecules secreted by pericardial fat could contribute to the coronary calcification [37, 38]. Overall, our findings support a relationship between the HDL-C/ApoA-I index with an adverse cardiometabolic profile and subclinical atherosclerosis that could be used to evaluate cardiovascular disease in adults without CAD.

### Strengths and limitations

We have some strengths and limitations to acknowledge. Compared to similar studies, our sample size is larger and better characterized from the demographic, clinical, and biochemical points of view. We included adult men and women of almost all ages, without a personal or family history of CAD and without diabetes mellitus, which reduces the bias in the interpretation of the associations, reducing the chance for confounding factors directly associated with the concentration, size, and function of HDL. This study contributes information to evaluate the utility of the HDL-C/ApoA-I ratio associated with subclinical atherosclerosis assessed by coronary artery calcium score in adults with asymptomatic CAD. Currently, the levels of HDL-C and ApoA-I are measured with very good precision and accuracy in all clinical laboratories, so the use of the HDL-C/ApoA-I index would provide additional information to estimate the probability of subclinical atherosclerosis. Nevertheless, the study has some limitations. This sub-analysis was carried out using the cross-sectional recruitment of the GEA study and does not allow for the establishment of the cause-effect relationship between the observed associations. The HDL sub-fractions were not directly analysed, and a precise relationship between HDL size and CAC score may not be established. Our results agree with previous reports where the HDL-C/ApoA-I index and HDL diameter estimation were used as surrogate biomarkers of HDL size [17, 28, 31]. This study included volunteers living in the metropolitan area of Mexico City, and the results could not be applicable to all the population, however, the prevalence of classical risk factors for CAD is similar to that found in the National Survey of Health and Nutrition 2016 (ENSANUT MC 2016), a randomized study and representative of the country [39].

## Conclusion

The HDL-C/ApoA-I index is inversely associated with an adverse coronary risk profile and subclinical atherosclerosis in asymptomatic subjects for CAD. The prevalence of CAC > 0 HU is higher in subjects with HDL-C/ApoA-I index less than 0.28. Of note the association of these index with CAC score is independent of classical risk factors, visceral, hepatic, and pericardial adiposity. Overall, these findings suggest that the HDL-C/ApoA-I index is practical and useful for optimizing the probability of subclinical atherosclerosis in a general population without CAD.

### Electronic supplementary material

Below is the link to the electronic supplementary material.

Supplementary table 1: Correlation between HDL-C/ApoA-I index and cardiometabolic risk factors. Abbreviations: BMI = Body Mass Index; L/SAR = Liver to spleen attenuation ratio.

Supplementary table 2: Linear regression models to evaluate the association of HDL-C/ApoA-I index and cardiometabolic risk components. BMI: Body Mass Index, SBP: Systolic Blood Pressure, DBP: Diastolic Blood Pressure, TC: total cholesterol, LDL-C: low-density lipoprotein cholesterol, HDL-C: High density lipoprotein cholesterol, TG: Triglycerides and HOMA-IR: Homeostatic model of insulin resistance. VAF: Visceral Abdominal Fat, SAF: Subcutaneous Abdominal Fat, PCF: Pericardial Fat Volume. L/SAR: Liver to spleen attenuation ratio. All models were adjusted for age, sex, BMI, TG, LDL-C, HOMA-IR, L/SAR, physical activity, and smoking, except for the models in which these variables are included as outcomes.

Supplementary table 3: Logistic regression models to evaluate the association of quartiles of HDL-C/ApoA-I index and cardiometabolic risk factors. HOMA-IR: Homeostatic model of insulin resistance. VAF: Visceral Abdominal Fat, SAF: Subcutaneous Abdominal Fat and PCF: Pericardial Fat Volume, TG: triglycerides, HDL-C < 50 in women and < 40 in men, LDL-C: low density lipoprotein, NAFLD: nonalcoholic fatty liver disease, MS: Metabolic Syndrome. All models were adjusted for age, sex, BMI, TG, LDL-C, HOMA-IR, L/SAR, physical activity, and smoking, except for the models in which these variables are included as outcomes.

## Data Availability

The data that support the findings of this study are available from the project administrator but restrictions apply to the availability of these data, which were used under license for the current study, and so are not publicly available. Data are however available from the corresponding author upon reasonable request and with permission of the project administrator.

## Abbreviations

HDL-C/ApoA-I: Low high density lipoprotein cholesterol to apo A-I ratio
GEA: Genetics of Atherosclerotic Disease study
HDL: High density lipoprotein
CAC: Coronary Artery Calcium score
CAD: Coronary artery disease
BMI: Body Mass Index
SBP: Systolic Blood Pressure
DBP: Diastolic Blood Pressure
TC: Total Cholesterol
TG: Triglycerides
HDL-C: High density lipoprotein Cholesterol
ApoA-I: Apolipoprotein AI
LDL-C: Low Density Lipoprotein Cholesterol
HOMA-IR: Homeostatic Model of Insulin Resistance
T: Tomography
TAF: Total Abdominal Fat
SAF: Subcutaneous Abdominal Fat
VAF: Visceral Abdominal Fat
PCF: Pericoronary Fat
NAFLD: Non-alcoholic fatty liver disease
HU: Hounsfield Units
BIC: Bayesian Information Criteria
L/SAR: Liver to Spleen Attenuation Ratio
NFκB: Factor Nuclear kappa B
ENSANUT: Encuesta Nacional de Salud y Nutrición
