# Supplementary material for: Low HDL-C/ApoA-I index is associated with cardiometabolic risk factors and coronary artery calcium: a sub-analysis of the genetics of atherosclerotic disease (GEA) study
Source: BMC Endocr Disord. 2024 Jul 11;24:110. doi: 10.1186/s12902-024-01642-0 (PMC11238479; doi:10.1186/s12902-024-01642-0)
Supplement: Supplementary file 1 — Supplementary table 1: Correlation between HDL-C/ApoA-I index and cardiometabolic risk factors. Abbreviations: BMI = Body Mass Index; L/SAR = Liver to spleen attenuation ratio. [file 12902_2024_1642_MOESM1_ESM.docx]

**Supplementary Table 2:** Linear regression models to evaluate the association of HDL-C/ApoA-I index and cardiometabolic risk components.

| Outcome | Predictor | Beta coefficient | 95% CI | p-value |
| --- | --- | --- | --- | --- |
| Age, (years) | HDL-C/ApoA-I index | 12 | 5.7, 18 | <0.001 |
| Waist, (cm) |  | -30 | -38, -23 | <0.001 |
| BMI (kg/m2) |  | -11 | -14, -7.8 | <0.001 |
| SBP, (mmHg) |  | -17 | -28, -4.9 | 0.005 |
| DBP, (mmHg) |  | -13 | -20, -7.1 | <0.001 |
| TC (mmol/L) |  | 0.44 | -0.21, 1.1 | 0.2 |
| LDL-C (mmol/L) |  | -0.03 | -0.59, 0.53 | >0.9 |
| TG (mmol/L) |  | -6.1 | -6.9, -5.3 | <0.001 |
| HDL-C (mmol/L) |  | 2.8 | 2.6, 2.9 | <0.001 |
| Glucose (mmol/L) |  | -0.97 | -1.3, -0.62 | <0.001 |
| Insulin (IU/mL) |  | -3.7 | -4.7, -2.8 | <0.001 |
| HOMA-IR |  | -6.7 | -8.4, -5.0 | <0.001 |
| VAF (cm2) |  | -150 | -193, -107 | <0.001 |
| SAF (cm2) |  | -43 | -121, 34 | 0.3 |
| PCF (cm2) |  | -80 | -99, -61 | <0.001 |
| L/SAR |  | 0.66 | 0.49, 0.83 | <0.001 |

BMI: Body Mass Index, SBP: Systolic Blood Pressure, DBP: Diastolic Blood Pressure, TC: total cholesterol, LDL-C: low-density lipoprotein cholesterol, HDL-C: High density lipoprotein cholesterol, TG: Triglycerides and HOMA-IR: Homeostatic model of insulin resistance. VAF: Visceral Abdominal Fat, SAF: Subcutaneous Abdominal Fat, PCF: Pericardial Fat Volume. L/SAR: Liver to spleen attenuation ratio. All models were adjusted for age, sex, BMI, TG, LDL-C, HOMA-IR, L/SAR, physical activity, and smoking, except for the models in which these variables are included as outcomes.
