# Supplementary material for: Low HDL-C/ApoA-I index is associated with cardiometabolic risk factors and coronary artery calcium: a sub-analysis of the genetics of atherosclerotic disease (GEA) study
Source: BMC Endocr Disord. 2024 Jul 11;24:110. doi: 10.1186/s12902-024-01642-0 (PMC11238479; doi:10.1186/s12902-024-01642-0)
Supplement: Supplementary file 2 — Supplementary table 2: Linear regression models to evaluate the association of HDL-C/ApoA-I index and cardiometabolic risk components. BMI: Body Mass Index, SBP: Systolic Blood Pressure, DBP: Diastolic Blood Pressure, TC: total cholesterol, LDL-C: low-density lipoprotein cholesterol, HDL-C: High density lipoprotein cholesterol, TG: Triglycerides and HOMA-IR: Homeostatic model of insulin resistance. VAF: Visceral Abdominal Fat, SAF: Subcutaneous Abdominal Fat, PCF: Pericardial Fat Volume. L/SAR: Liver to spleen attenuation ratio. All models were adjusted for age, sex, BMI, TG, LDL-C, HOMA-IR, L/SAR, physical activity, and smoking, except for the models in which these variables are included as outcomes. [file 12902_2024_1642_MOESM2_ESM.docx]

**Supplementary Table 3:** Logistic regression models to evaluate the association of quartiles of HDL-C/ApoA-I index and cardiometabolic risk factors.

| Outcome | Quartiles of HDL-C/Apo-AI index | Odds Ratio | 95% CI | p-value |
| --- | --- | --- | --- | --- |
| HOMA-IR >p75 |  |  |  |  |
|  | Q 4 | — | — |  |
|  | Q 3 | 1.16 | 0.80, 1.68 | 0.4 |
|  | Q 2 | 0.92 | 0.63, 1.34 | 0.7 |
|  | Q 1 | 1.12 | 0.73, 1.72 | 0.6 |
| Arterial Hypertension |  |  |  |  |
|  | Q 4 | — | — |  |
|  | Q 3 | 1.27 | 0.74, 2.21 | 0.4 |
|  | Q 2 | 0.68 | 0.37, 1.26 | 0.2 |
|  | Q 1 | 0.91 | 0.49, 1.68 | 0.8 |
| VAF >p75 |  |  |  |  |
|  | Q 4 | — | — |  |
|  | Q 3 | 0.66 | 0.45, 0.96 | 0.031 |
|  | Q 2 | 0.96 | 0.65, 1.41 | 0.8 |
|  | Q 1 | 0.77 | 0.50, 1.19 | 0.2 |
| SAF >p75 |  |  |  |  |
|  | Q 4 | — | — |  |
|  | Q 3 | 0.83 | 0.54, 1.27 | 0.4 |
|  | Q 2 | 0.99 | 0.65, 1.51 | >0.9 |
|  | Q 1 | 0.80 | 0.50, 1.27 | 0.3 |
| PCF >p75 |  |  |  |  |
|  | Q 4 | — | — |  |
|  | Q 3 | 0.73 | 0.46, 1.16 | 0.2 |
|  | Q 2 | 0.97 | 0.62, 1.54 | >0.9 |
|  | Q 1 | 0.65 | 0.39, 1.08 | 0.10 |
| Overweight |  |  |  |  |
|  | Q 4 | — | — |  |
|  | Q 3 | 1.16 | 0.85, 1.58 | 0.4 |
|  | Q 2 | 1.58 | 1.15, 2.18 | 0.005 |
|  | Q 1 | 1.33 | 0.94, 1.88 | 0.11 |
| Obesity |  |  |  |  |
|  | Q 4 | — | — |  |
|  | Q 3 | 1.28 | 0.86, 1.90 | 0.2 |
|  | Q 2 | 1.07 | 0.72, 1.60 | 0.7 |
|  | Q 1 | 1.19 | 0.78, 1.82 | 0.4 |
| TG > 150 mg/dl |  |  |  |  |
|  | Q 4 | — | — |  |
|  | Q 3 | 1.81 | 1.28, 2.57 | <0.001 |
|  | Q 2 | 3.75 | 2.66, 5.33 | <0.001 |
|  | Q 1 | 7.31 | 5.03, 10.7 | <0.001 |
| Low HDL-C |  |  |  |  |
|  | Q 4 | — | — |  |
|  | Q 3 | 1.87 | 1.32, 2.67 | <0.001 |
|  | Q 2 | 3.80 | 2.67, 5.44 | <0.001 |
|  | Q 1 | 8.40 | 5.60, 12.7 | <0.001 |
| LDL > 100 mg/dl |  |  |  |  |
|  | Q 4 | — | — |  |
|  | Q 3 | 1.57 | 1.10, 2.24 | 0.014 |
|  | Q 2 | 1.07 | 0.76, 1.53 | 0.7 |
|  | Q 1 | 0.71 | 0.49, 1.03 | 0.072 |
| NAFLD |  |  |  |  |
|  | Q 4 | — | — |  |
|  | Q 3 | 1.17 | 0.80, 1.72 | 0.4 |
|  | Q 2 | 1.30 | 0.89, 1.90 | 0.2 |
|  | Q 1 | 1.72 | 1.15, 2.58 | 0.008 |
| MS |  |  |  |  |
|  | Q 4 | — | — |  |
|  | Q 3 | 1.50 | 0.97, 2.34 | 0.068 |
|  | Q 2 | 1.97 | 1.28, 3.06 | 0.002 |
|  | Q 1 | 3.10 | 1.97, 4.94 | <0.001 |

HOMA-IR: Homeostatic model of insulin resistance. VAF: Visceral Abdominal Fat, SAF: Subcutaneous Abdominal Fat and PCF: Pericardial Fat Volume, TG: triglycerides, HDL-C <50 in women and <40 in men, LDL-C: low density lipoprotein, NAFLD: nonalcoholic fatty liver disease, MS: Metabolic Syndrome. All models were adjusted for age, sex, BMI, TG, LDL-C, HOMA-IR, L/SAR, physical activity, and smoking, except for the models in which these variables are included as outcomes.
