# Supplementary material for: Low HDL-C/ApoA-I index is associated with cardiometabolic risk factors and coronary artery calcium: a sub-analysis of the genetics of atherosclerotic disease (GEA) study
Source: BMC Endocr Disord. 2024 Jul 11;24:110. doi: 10.1186/s12902-024-01642-0 (PMC11238479; doi:10.1186/s12902-024-01642-0)
Supplement: Supplementary file 3 — Supplementary table 3: Logistic regression models to evaluate the association of quartiles of HDL-C/ApoA-I index and cardiometabolic risk factors. HOMA-IR: Homeostatic model of insulin resistance. VAF: Visceral Abdominal Fat, SAF: Subcutaneous Abdominal Fat and PCF: Pericardial Fat Volume, TG: triglycerides, HDL-C < 50 in women and < 40 in men, LDL-C: low density lipoprotein, NAFLD: nonalcoholic fatty liver disease, MS: Metabolic Syndrome. All models were adjusted for age, sex, BMI, TG, LDL-C, HOMA-IR, L/SAR, physical activity, and smoking, except for the models in which these variables are included as outcomes. [file 12902_2024_1642_MOESM3_ESM.docx]

**Supplementary Table 1** Correlation between HDL-C/ApoA-I index and cardio-

metabolic risk factors.

|  | HDL-C/ApoA-I index | | | |
| --- | --- | --- | --- | --- |
| Variable | Spearman rho | 95% CI | p-value |  |
| Age (years) | 0.072 | 0.0191 to - 0.124 | 0.008 |  |
| Waist circumference (cm) | -0.224 | -0.273 to -0.172 | <0.001 |  |
| BMI (kg/m²) | -0.222 | -0.272 to -0.171 | <0.001 |  |
| Systolic blood pressure (mmHg) | -0.093 | -0.145 to -0.039 | 0.001 |  |
| Diastolic blood pressure (mmHg) | -0.112 | -0.163 to -0.058 | <0.001 |  |
| Total cholesterol (mmol/L) | 0.042 | -0.011 to 0.094 | 0.121 |  |
| LDL-C (mmol/L) | -0.013 | -0.065 to 0.040 | 0.64 |  |
| Triglycerides (mmol/L) | -0.437 | -0.478 to -0.392 | <0.001 |  |
| HDL-C (mmol/L) | 0.596 | 0.560 to 0.629 | <0.001 |  |
| Glucose (mmol/L) | -0.131 | -0.182 to -0.078 | <0.001 |  |
| Insulin (μU/mL) | -0.219 | -0.269 to -0.167 | <0.001 |  |
| HOMA-IR (Homeostasis Model  Assessment of Insulin Resistance) | -0.225 | -0.275 to -0.173 | <0.001 |  |
| Apo A1 (mmol/L) | -0.193 |  | <0.001 |  |
| Total abdominal fat area (cm²) | 0.895 | -0.161 to -0.055 | <0.001 |  |
| Visceral fat (cm²) | -0.109 | -0.271 to -0.168 | <0.001 |  |
| Subcutaneous fat (cm²) | -0.027 | -0.080 to 0.026 | 0.318 |  |
| Pericardial fat (cm^2^) | -0.228 | -0.344 to -0.228 | <0.001 |  |
| L/SAR | 0.212 | 0.159 to 0.262 | <0.001 |  |

Abbreviations: BMI= Body Mass Index; L/SAR= Liver to spleen attenuation ratio.
